# Supplementary material for: Comparative multiomics analysis of cell physiological state after culture in a basket bioreactor
Source: Sci Rep. 2022 Nov 23;12:20161. doi: 10.1038/s41598-022-24687-4 (PMC9686226; doi:10.1038/s41598-022-24687-4)

3666051.1:32779936:32781072:+@NW_023666051.1:32782391:32782532:+@NW_023666051.1:32782394:3278

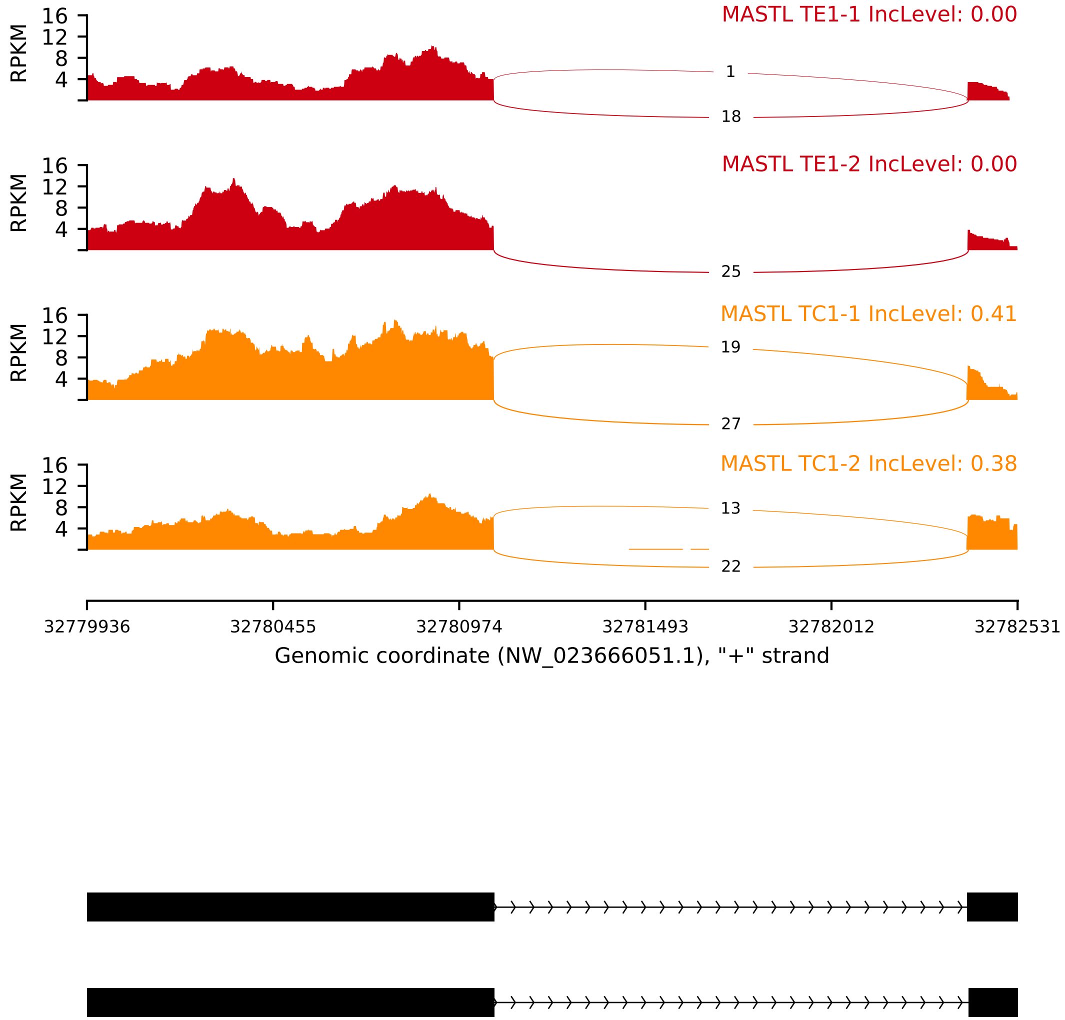

Supplement: Supplementary file 1 — Supplementary Information 1. [file 41598_2022_24687_MOESM1_ESM.zip › raw data/Transcriptomic raw data/7.AS/2.dasplot/TE1vsTC1/A3SS/6_MASTL_NW_023666051.1_32779936_32781072_+@NW_023666051.1_32782391_32782532_+@NW_023666051.1_32782394_32782532_+.pdf]
